# Evaluation of the Anatomical Reference Point in Posterior Minimally Invasive Atlantoaxial Spine Surgery: A Cadaveric Anatomical Study

**DOI:** 10.1111/os.14023

**Published:** 2024-03-04

**Authors:** Peirong Lian, Hu Chen, Wanshun Wang, Changrong Zhu, Qiang Tu, Xiangyang Ma, Hong Xia, Honglei Yi

**Affiliations:** ^1^ The First School of Clinical Medicine Southern Medical University Guangzhou China; ^2^ Department of Orthopaedic People's Liberation Army General Hospital of Southern Theatre Command Guangzhou China; ^3^ The Second Clinical Medical College Guangzhou University of Chinese Medicine Guangzhou China

**Keywords:** Atlantoaxial Surgery, Minimally Invasive, Posterior, Reference Points

## Abstract

**Objective:**

Minimally invasive atlantoaxial surgery offers the benefits of reduced trauma and quicker recovery. Previous studies have focused on feasibility and technical aspects, but the lack of comprehensive safety information has limited its availability and widespread use. This study proposes to define the feasibility and range of surgical safety using the intersection of the greater occipital nerve and the inferior border of the inferior cephalic oblique as a reference point.

**Methods:**

Dissection was performed on 10 fresh cadavers to define the anatomical reference point as the intersection of the greater occipital nerve and the inferior border of the inferior cephalic oblique muscle. The study aimed to analyze the safety range of minimally invasive atlantoaxial fusion surgery by measuring the distance between the anatomical reference point and the transverse foramen of the axis, the distance between the anatomical reference point and the superior border of the posterior arch of the atlas, and the distance between the anatomical reference point and the spinal canal. Measurements were compared using Student's t test.

**Results:**

The point where the occipital greater nerve intersects with the inferior border of the inferior cephalic oblique muscle was defined as the anatomical marker for minimally invasive posterior atlantoaxial surgery. The distance between this anatomical marker and the transverse foramen of the axis was measured to be 9.32 ± 2.04 mm. Additionally, the distance to the superior border of the posterior arch of the atlas was found to be 21.29 ± 1.93 mm, and the distance to the spinal canal was measured to be 11.53 ± 2.18 mm. These measurement results can aid surgeons in protecting the vertebral artery and dura mater during minimally invasive posterior atlantoaxial surgery.

**Conclusions:**

The intersection of the greater occipital nerve with the inferior border of the inferior cephalic oblique muscle is a safe and reliable anatomical landmark in minimally invasive posterior atlantoaxial surgery.

## Graphical abstract

The intersection of the greater occipital nerve with the inferior border of the inferior cephalic oblique muscle and the muscles was revealed during dissection, including trapezius, splenius capitis, semispinalis capitis, suboccipital triangle.

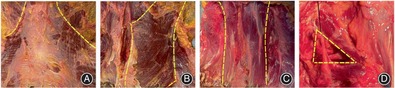

## Introduction

Atlantoaxial dislocations can result in significant neurological deficits and typically necessitate surgical intervention. Posterior atlantoaxial fusion is currently the mainstay of treatment for atlantoaxial dislocations, as it offers dependable internal fixation, appropriate repositioning forces, and facilitates atlantoaxial stabilization and bone graft fusion. However, the conventional surgical technique of posterior C1–C2 fusion requires stripping the suboccipital musculature from the midline to expose the entry point for the C1 lateral mass screw and the C2 pedicle screw. This approach often leads to significant damage to the muscles attached to the C2 spinous process, as well as soft tissues such as the myodural bridge and collateral ligaments. Consequently, patients may experience postoperative axial pain, with reported rates ranging from 7% to 58%.^1^ Research has shown that cervical muscle strength is associated with axial symptoms^2^ and preservation of the muscles attached to the C2 spinous process may potentially reduce the incidence of axial pain.^3^, ^4^ In addition, minimally invasive atlantoaxial surgery offers a significant number of advantages, including protection of the suboccipital muscles, reduced bleeding, shorter hospital stay, reduced reliance on pain medication, and no significant increase in complications.^5^ Recent studies have reported minimally invasive atlantoaxial fusion procedures performed percutaneously using intraoperative CT navigation. However, it should be noted that the percutaneous technique is entirely dependent on intraoperative CT navigation. It has not yet been widely adopted.^6^, ^7^ The remaining studies all focused on minimally invasive atlantoaxial surgery performed under direct visualization. Anatomical studies of minimally invasive atlantoaxial fusion surgery have been reported since 2006^8^, including minimally invasive access through a paramedian incision^9^, ^10^, ^11^ and a posterior bilateral musculocutaneous approach through a median incision^5^, ^12^, ^13^ to complete the atlantoaxial fusion. Both approaches were performed with direct visualization, which did not require intraoperative CT navigation.

However, complex craniocervical anatomy limits adoption of minimally invasive atlantoaxial fusion techniques under direct vision. While previous studies have focused on feasibility and technical aspects, they have often neglected to address the safety considerations of minimally invasive surgery, particularly regarding the avoidance of potential injuries to critical structures such as the vertebral artery and the dura mater. The limited availability and widespread adoption of direct vision minimally invasive atlantoaxial surgery may be significantly influenced by the lack of comprehensive safety information.

The suboccipital triangle is a crucial anatomical structure in minimally invasive atlantoaxial fusion procedures. It is defined by the superior cephalic oblique, inferior cephalic oblique, and posterior cephalic greater rectus muscles, and it encompasses important structures such as the dura mater and the V3 segment of the vertebral artery.^14^, ^15^, ^16^ The occipital greater nerve emerges from the dorsal branch of the C2 nerve, passes medially from the inferior edge of the inferior cephalic oblique muscle, traverses obliquely through the suboccipital triangle, and then extends laterally along the posterior cephalic greater rectus muscle. The diameter of the occipital greater nerve can reach up to 3.5 mm, and it has a close relationship with the inferior cephalic oblique muscle.^17^, ^18^, ^19^ Consequently, the intersection of the occipital greater nerve and the inferior edge of the inferior cephalic oblique muscle is a significant point of interest in these procedures.

The purpose of this study was to: (i) confirm the anatomical reference points in posterior minimally invasive atlantoaxial spine surgery; (ii) measure the relative position of the anatomical reference point to important anatomical structures within the minimally invasive surgical field; and (iii) clarify the safe range of minimally invasive atlantoaxial surgery.

## Materials and Methods

This study was conducted in accordance with the Declaration of Helsinki and approved by the institutional review committee of People's Liberation Army (PLA) General Hospital of Southern Theater Command (approval number: 20220457).

### Study Design

Ten fresh cadavers were dissected to reveal the intersection of the greater occipital nerve with the inferior border of the inferior cephalic oblique muscle, as well as the optimal placement points for the C1 lateral block screw and the C2 pedicle screw while preserving the inferior occipital muscle group. Kerf pins were carefully fixed to the bone surfaces at each identified location using an electric drill. The intersection of the greater occipital nerve with the inferior border of the inferior cephalic oblique muscle was used as the anatomical marker point. The distance between this marker point and the surrounding important anatomical structures was meticulously measured and detailed photographs were taken to record each anatomical structure. After the dissection was completed, the specimen underwent CT scanning, and the CT images were reconstructed using Mimics 21.0 software (Materialize Company, Belgium). This allowed for a re‐evaluation of the anatomical parameters associated with the bony structures. The relative position of the anatomical reference points to each anatomical structure was assessed, and the safe operating range of minimally invasive atlantoaxial fusion was clarified through these measurements and evaluations.

### Anatomical Steps and Measurement Parameters

**FIGURE 1:**
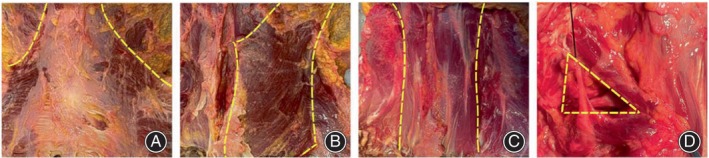
The muscles revealed during dissection, including trapezius (A), splenius capitis (B), semispinalis capitis (C), suboccipital triangle (D).

The cadaver was placed in prone position, a horizontal incision was made along the inferior collar line and a vertical incision was made along the median line to free the skin and fascia. The trapezius muscle was lifted away from the occipital bone, moving outward and downward. The splenius capitis were then longitudinally separated, while the semispinalis capitis were transversely separated. These steps were taken to expose and reveal the structures including inferior cephalic oblique, superior cephalic oblique, posterior cephalic major rectus, and posterior cephalic minor rectus (Figure 1).

**FIGURE 2:**
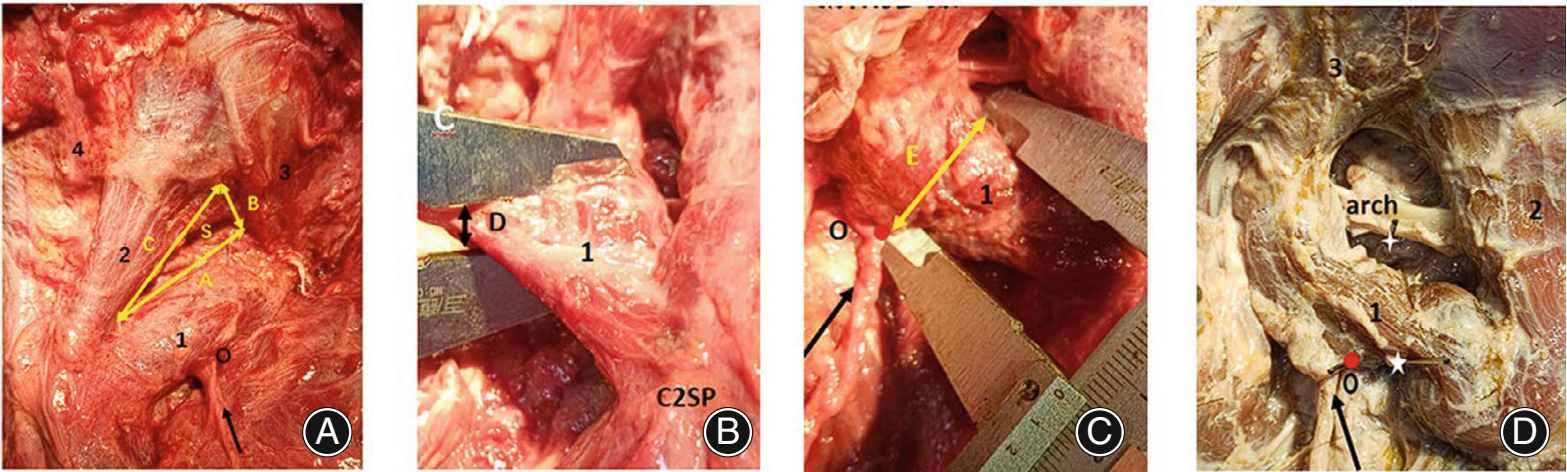
The measurement of muscle‐related anatomical parameters and marked anatomical reference point (O), C1 lateral mass screw placement points and C2 pedicle screw placement points with Kirschner pins while preserving the inferior occipital triangle. (1) inferior cephalic oblique, (2) posterior cephalic major rectus, (3) superior cephalic oblique, (4) posterior cephalic minor rectus. (A) The anatomical structures and measurement parameters related to the inferior occipital triangle, measuring the length of the superior border of the inferior cephalic oblique (A), the length of the inner border of the superior cephalic oblique (B), the length of the outer border of the posterior cephalic greater rectus (C), area of the suboccipital triangle (S); (B) measure the thickness of the inferior cephalic oblique at the anatomical reference point (D); (C) measure the width of the inferior cephalic oblique muscle at the anatomical reference point (E); (D) show the bony structures marked by the kerf pins, arch represents C1 posterior arch, red circle represents the intersection of the great occipital nerve with the inferior border of the inferior cephalic oblique, white four‐pointed star represents C1 lateral mass screw placement point, and white five‐pointed star represents C2 pedicle screw placement point.

The length of the inner border of the suboccipital triangle, which includes the length of the superior border of the inferior cephalic oblique (A), the length of the inner border of the superior cephalic oblique (B), and the length of the outer border of the posterior cephalic greater rectus (C), was measured with vernier calipers. The area of the suboccipital triangle (S) was calculated based on these measurements. The relationship between the great occipital nerve and the inferior cephalic oblique was observed. The point where the great occipital nerve intersected with the inferior border of the inferior cephalic oblique was marked using a kerchief needle. This point, marked by the kerchief needle and fixed with an electric drill on the C2 vertebral plate, was defined as the anatomical reference point (O). The thickness (D) and width (E) of the inferior cephalic oblique at the anatomic reference point were measured. The soft tissue of the suboccipital triangle was then dissected, revealing the posterior arch of C1 and the atlantoaxial joint within the interspace between the posterior rectus major and inferior cephalic oblique muscles. A kerchief needle was drilled at the C1 lateral mass screw placement site, and the inferior cephalic oblique muscle was pulled upward. This allowed for the visualization of the C2 vertebral plate beneath the inferior cephalic oblique, and a kerchief needle was drilled at the C2 pedicle screw placement site (Figure 2).

**FIGURE 3:**
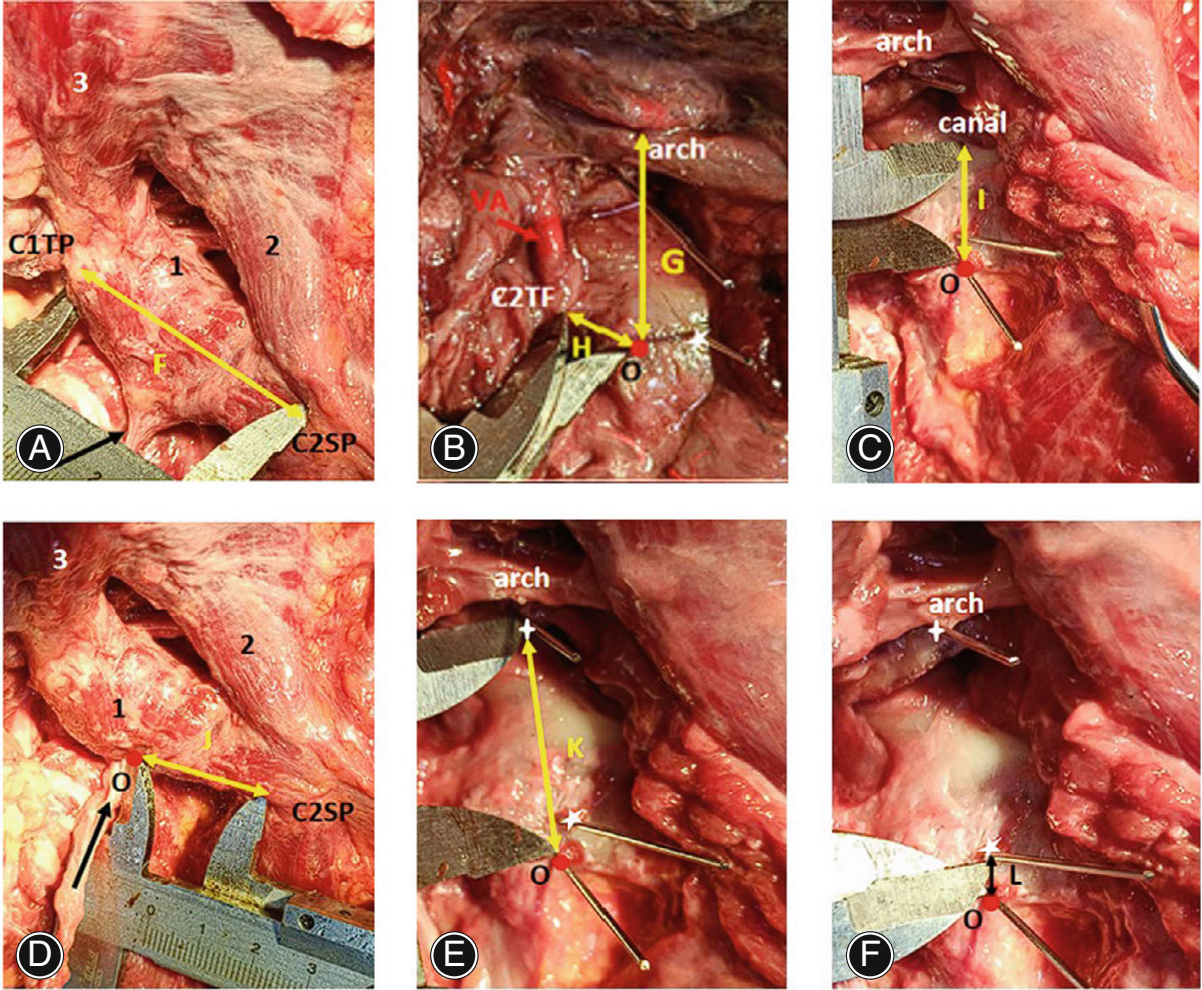
The measurement of bony anatomical parameters. (1) Inferior oblique muscle, (2) posterior rectus major, (3) superior oblique muscle, C1TP for C1 transverse foramen, C2SP for C2 spinous process, C2TF for C2 transverse foramen, red circle for anatomical reference point (O), and canal for the spinal canal. (A) The distance from C2 spinous process to C1 transverse process (F); (B) the distance from the anatomical reference point to the superior edge of the posterior arch of the atlas (G), the anatomical reference point to the C2 transverse foramen (H); (C) the distance from the anatomical reference point to the canal (I); (D) the distance from the anatomical reference point to the C2 spinous process (J); (E) the distance from the anatomical reference point to the C1 lateral mass screw placement point (K); (F) the distance from the anatomical reference point to the C2 pedicle screw placement point (L).

**FIGURE 4:**
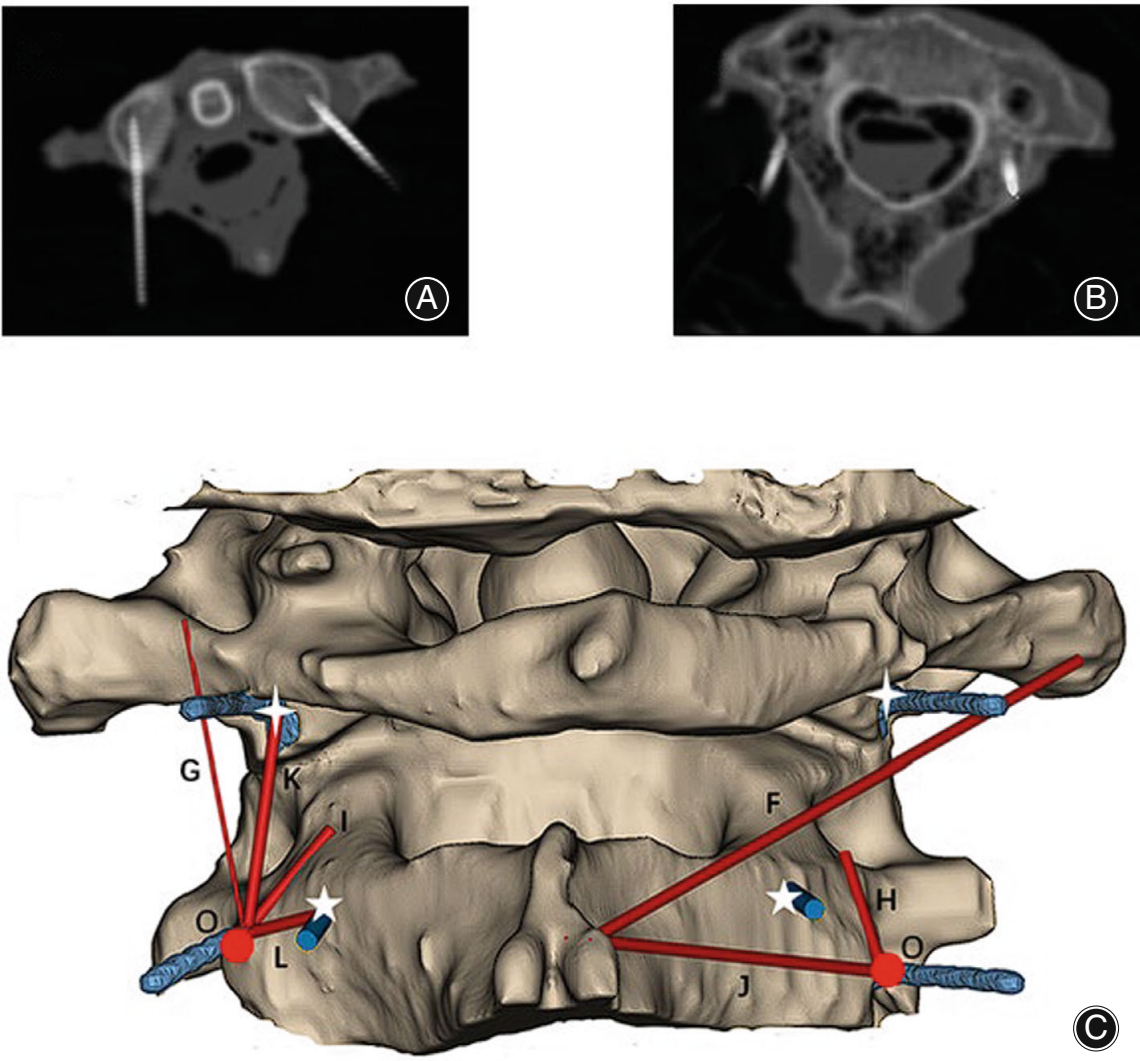
Axial CT scans and Mimics reconstruction images, red circles represent the anatomical reference point (O) marked by the kerf pins, white four‐pointed stars represent the C1 lateral mass screw placement points marked by the kerf pins, and white five‐pointed stars represent the C2 pedicle screw placement points marked by the kerf pins. (A) Axial CT shows the C1 lateral mass screw placement points marked by the kerf pins bilaterally; (B) the distance from C2 spinous process to C1 transverse process (F), the distance from the anatomical reference point to the superior edge of the posterior arch of the atlas (G), the distance from the anatomical reference point to the transverse foramen of the axis (H), the distance from the anatomical reference point to the spinal canal (I), the distance from the anatomical reference point to C2 spinous process (J), the distance from the anatomical reference point to C1 lateral mass screws (K), and the distance from the anatomical reference point to C2 pedicle screws (L) were measured.

The bony anatomical parameters were measured at the anatomical reference point, including the distance from the C1 transverse process to the C2 spinous process (F), the distance from the anatomical reference point to the superior edge of the C1 posterior arch (G), the distance from the anatomical reference point to the medial edge of the C2 transverse foramen (H), the distance from the anatomical reference point to the spinal canal (I), the distance from the anatomical reference point to the C2 spinous process (J), the distance from the anatomical reference point to the C1 lateral mass screw entry point (K), and the distance from the anatomical reference point to the C2 pedicle screw entry point (L), and the distance from the anatomical reference point to the C2 pedicle screw placement point (L) (Figure 3). After these measurements were taken, the specimen underwent CT scanning. The CT images were then reconstructed using Mimics 21.0 software. The bony anatomical parameters were re‐measured on the 3D reconstructed CT images to ensure accuracy and obtain detailed information (Figure 4).

### Illustrative Case

**FIGURE 5:**
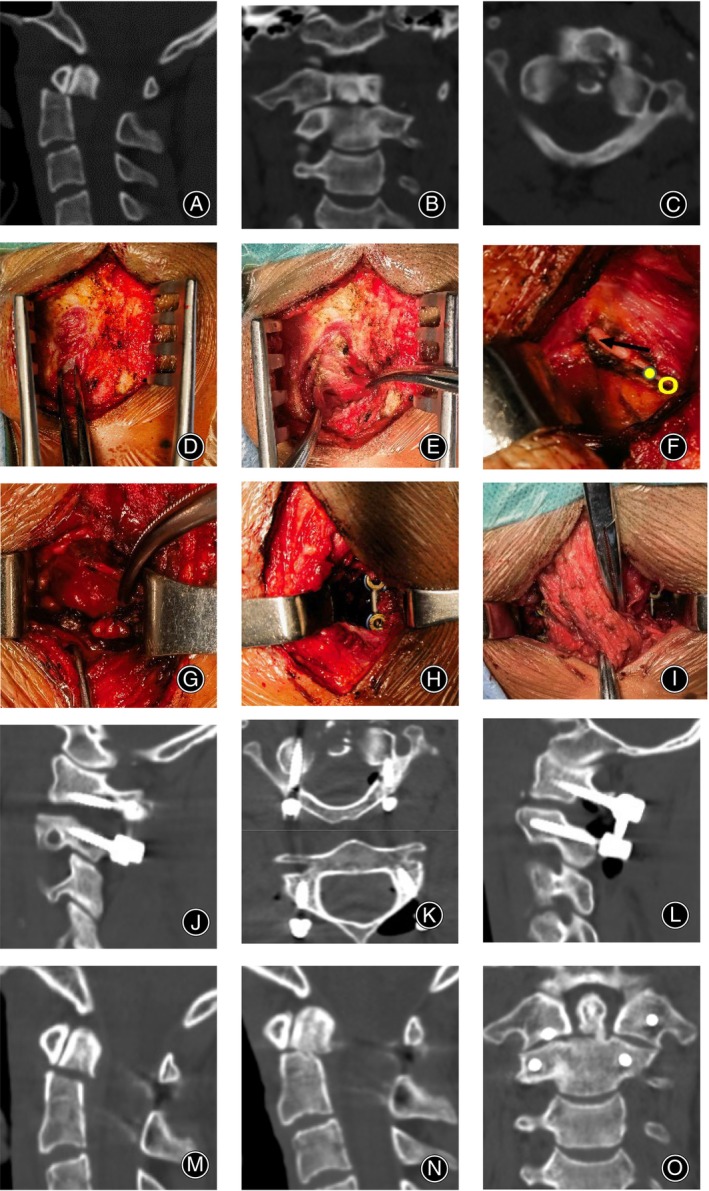
An illustrative case of posterior minimally invasive atlantoaxial repositioning and internal fixation. A male patient, aged 30, presented with post‐traumatic neck pain and discomfort. A preoperative cervical spine CT scan suggested an odontoid fracture (A–C), and surgery was performed. During the surgery, the surgeons reached the C2 spinous process and then split the trapezius muscle fibers 2 cm lateral to it (D). The study involved separating the splenius capitis and semispinalis capitis muscles from their bellies (E) to reveal the intersection of the greater occipital nerve and the inferior border of the inferior cephalic oblique, which served as the anatomical reference point. The exposure was performed along the inferior border of the inferior cephalic oblique (G–I) medial to this point. Finally, the atlantoaxial internal fixation screws were placed. Postoperative imaging showed that the atlantoaxial internal fixation was in a good position and the odontoid was well repositioned (J–M). A follow‐up CT scan conducted 3 months after the surgery revealed that the patient's odontoid fracture had started to heal (N, O).

A 30‐year‐old male was diagnosed as odontoid fracture (Figure 5A–C) and underwent posterior minimally invasive atlantoaxial repositioning and internal fixation. The patient was placed in the prone position after general anesthesia via tracheal intubation, immobilized using a Mayfield skull clamp, and the skin was incised along the midline to release the fascia bilaterally. The study first reached the C2 spinous process and then split the trapezius muscle fibers 2 cm lateral to it (Figure 5D). The splenius capitis and semispinalis capitis muscles were then separated from their bellies (Figure 5E), revealing the intersection of the greater occipital nerve and the inferior border of the inferior cephalic oblique, which serves as the anatomical reference point in this study (Figure 5F). Exposure was performed along the inferior border of the inferior cephalic oblique (Figure 5G–I) medial to this point. This approach avoids damaging the vertical portion of the V3 segment of the vertebral artery, which is lateral to the anatomic reference point. Additionally, it can prevent injury to the horizontal portion of the V3 segment of the vertebral artery and the dura mater above the anatomical reference point. Finally, the placement of the atlantoaxial internal fixation screws was accomplished using C‐arm fluoroscopy. The contralateral internal fixation was also accomplished in the same manner. The operation lasted 120 min with 50 ml of intraoperative bleeding and no surgical complications. Postoperative imaging indicated that the atlantoaxial internal fixation was in a good position and the odontoid was well repositioned (Figure 5J–M). A follow‐up CT scan conducted 3 months after the surgery revealed that the patient's odontoid fracture had started to heal (Figure 5N, O).

### Statistical Analysis

Each measurement was performed three times and the average value was calculated. The results were then presented as mean ± standard deviation. The measurement data were analyzed using SPSS 27 (IBM Corporation, USA), and paired t‐test were performed on the bilateral data measured by cadaver specimens, the bilateral data measured by Mimics 21.0 software, and the data measured by cadaver specimens and Mimics 21.0 software. A significance level of *p* ≤ 0.05 was considered to indicate a statistically significant difference.

## Result

### Muscle‐Related Anatomical Parameters

The measurements of muscle‐related anatomical parameters are shown in Table 1.The average length of the superior border of the inferior cephalic oblique muscle was 29.40 ± 4.70 mm, the average length of the medial border of the superior cephalic oblique muscle was 16.76 ± 2.37 mm, and the average length of the external border of the posterior rectus major muscle was 30.67 ± 3.19 mm. The average area of the suboccipital triangle was computed to be 2.38 ± 0.59 cm^2^. The intersection of the great occipital nerve with the inferior border of the inferior cephalic oblique muscle was used as the anatomical reference point. The average thickness of the inferior cephalic oblique at the anatomical reference point was 5.81 ± 1.78 mm and the average width here was 12.18 ± 2.30 mm.

**TABLE 1:** Measurements of muscle‐related parameters in cadaveric.

Measurement parameters	Variable	Left	Right	Total	*p* value
The length of the superior border of the inferior cephalic oblique (mm)	A	28.78 ± 4.63	30.02 ± 4.92	29.40 ± 4.70	0.298
The length of the inner border of the superior cephalic oblique (mm)	B	16.29 ± 3.11	17.22 ± 1.30	16.76 ± 2.37	0.388
The length of the outer border of the posterior cephalic greater rectus (mm)	C	30.54 ± 3.28	30.79 ± 3.26	30.67 ± 3.19	0.763
Area of the suboccipital triangle (cm^2^)	S	2.29 ± 0.69	2.46 ± 0.49	2.38 ± 0.59	0.354
The thickness of the inferior cephalic oblique at the anatomical reference point (mm)	D	5.85 ± 2.11	5.77 ± 1.51	5.81 ± 1.78	0.84
The width of the inferior cephalic oblique muscle at the anatomical reference point (mm)	E	12.16 ± 2.43	12.20 ± 2.30	12.18 ± 2.30	0.856

### Bony Anatomical Parameters

The results of the cadaveric measurements of the bony anatomical parameters and the results of the Mimics software 3D reconstruction measurements are shown in Table 2. The mean distance from C2 spinous process to C1 transverse process was 47.66 ± 4.76 mm. The mean distance from the anatomical reference point to the superior edge of the posterior arch of C1 was 21.29 ± 1.93 mm. The average distance from the anatomical reference point to the medial edge of the C2 transverse foramen was 9.32 ± 2.04 mm. The mean distance from the anatomical reference point to the spinal canal was 11.53 ± 2.18 mm. The mean distance from the anatomical reference point to the C2 spinous process was 25.09 ± 3.53 mm. The average distance from the anatomical reference point to the C1 lateral mass screw placement point was 18.13 ± 3.30 mm. The mean distance from the anatomic reference point to the C2 pedicle screw placement point was 4.23 ± 1.69 mm. The above bony anatomical parameters describe the relative position between the intersection of the great occipital nerve and the inferior border of the inferior cephalic oblique muscle and each anatomical structure.

**TABLE 2:** Measurements of bony parameters on cadaveric and Mimics three‐dimensional reconstructed images.

Measurement parameters	Variable	Cadaveric			Mimics			Cadaveric	Mimics	
		Left	Right	*p* value	Left	Right	*p* value	Total	Total	*p* value
The distance from C2 spinous process to C1 transverse process (mm)	F	47.37 ± 5.12	47.94 ± 4.63	0.671	48.34 ± 2.93	48.37 ± 2.72	0.969	47.66 ± 4.76	48.35 ± 2.75	0.35
The distance from the anatomical reference point to the superior edge of the posterior arch of C1 (mm)	G	21.13 ± 2.42	21.44 ± 1.40	0.713	21.55 ± 1.74	21.14 ± 1.66	0.473	21.29 ± 1.93	21.34 ± 1.67	0.848
The distance from the anatomical reference point to the C2 transverse foramen (mm)	H	10.06 ± 2.19	8.57 ± 1.65	0.151	9.64 ± 2.09	8.65 ± 1.41	0.28	9.32 ± 2.04	9.14 ± 1.81	0.527
The distance from the anatomical reference point to the canal (mm)	I	12.33 ± 2.08	10.73 ± 2.07	0.123	11.70 ± 1.81	10.56 ± 2.18	0.099	11.53 ± 2.18	11.13 ± 2.04	0.095
The distance from the anatomical reference point to the C2 spinous process (mm)	J	25.99 ± 4.41	24.18 ± 2.24	0.191	25.86 ± 2.39	25.20 ± 1.84	0.116	25.09 ± 3.53	25.53 ± 2.10	0.349
The distance from the anatomical reference point to the C1 lateral mass screw placement point (mm)	K	19.16 ± 3.19	17.10 ± 3.23	0.114	18.45 ± 2.54	17.05 ± 2.75	0.206	18.13 ± 3.30	17.75 ± 2.67	0.126
The distance from the anatomical reference point to the C2 pedicle screw placement point (mm)	L	4.23 ± 1.72	4.23 ± 1.76	1	4.19 ± 1.43	4.04 ± 0.99	0.604	4.23 ± 1.69	4.11 ± 1.20	0.471

There was no statistical difference between the bilateral data measured in the cadaveric and Mimics 3D reconstructed images, and no statistical difference between the cadaveric specimens and the Mimics data (Table 2).

## Discussion

This study clarifies the intersection of the greater occipital nerve and the inferior border of the inferior cephalic oblique muscle as an anatomical reference point in minimally invasive atlantoaxial surgery. It also measures the safety range of minimally invasive atlantoaxial surgery, which can assist surgeons in avoiding injury to the vertebral artery and dura mater during the operation.

### Development of Minimally Invasive Atlantoaxial Surgery

Traditional atlantoaxial fusion was performed through a posterior median cervical incision, where the skin and fascia were dissected. Subsequently, subperiosteal stripping of various muscles, including the trapezius, splenius capitis, semispinalis capitis, posterior cephalic major rectus, posterior cephalic minor rectus, and inferior cephalic oblique was carried out bilaterally from the midline. However, this approach can result in soft tissue injury, leading to significant postoperative pain, muscle dysfunction, and potentially increasing the risk of postoperative sagittal plane deformity and adjacent segmental disease.^20^ Research had indicated that preserving the muscles attached to the C2 spinous process can help reduce postoperative axial pain.^3^ Additionally, it had been reported that the musculodural bridge is connected between the posterior cephalic major rectus, posterior cephalic minor rectus, and inferior cephalic oblique muscles and the epidura, and the fibers of the musculodural bridge are fused into the epidura, which effectively transmits tension and pull to the epidura during contraction or relaxation of the suboccipital muscles.^21^ Once the suboccipital triangle is exposed, the C1 lateral mass screw and the C2 pedicle screw placement points can be accessed while preserving the integrity of the inferior occipital musculature. In contrast to traditional surgery, this approach allows for complete preservation of the posterior cephalic major rectus, posterior cephalic minor rectus, and inferior cephalic oblique muscles, without compromising the musculodural bridge connecting the inferior occipital musculature to the epidural space. Clinical studies had indicated that minimally invasive atlantoaxial fusion surgery can result in reduced perioperative bleeding and postoperative pain.^5^ However, further research is necessary to establish the safety and efficacy of minimally invasive approaches for atlantoaxial surgery.

In this study, a layer‐by‐layer dissection was performed along the minimally invasive surgical approach, starting with the trapezius muscle and progressing to the splenius capitis and semispinalis capitis. To optimize visualization of the suboccipital triangle, the superficial layers including the splenius capitis and semispinalis capitis were removed. Previously reported minimally invasive posterior atlantoaxial fusion procedures  involved two different approaches. One approach involved utilizing a minimally invasive channel created through bilateral incisions approximately 2 cm from the midline. This channel traversed through the trapezius, splenius capitis, semispinalis capitis, and inferior cephalic oblique muscles, allowing for anchoring to the C2 lamina. By gradually expanding the channel, the C1 lateral mass screws and C2 pedicle screw placement points could be accessed, enabling completion of the atlantoaxial internal fixation.^9^, ^10^, ^11^ The advantage of this approach is that it involves a small surgical incision and reaches the target point by passing through the muscle belly, minimizing disruption to the splenius capitis and semispinalis capitis muscle fibers. However, this approach provides a limited surgical field of view and often relies on expandable channels and microscopic instruments. Additionally, it requires bilateral incisions to complete the procedure. Another minimally invasive surgical approach involved dissecting the trapezius, splenius capitis, and semispinalis capitis muscles from appropriate positions by making a midline incision through the skin and fascia. This allowed for exposure of the C1 lateral mass and C2 lamina through the muscle gap between the inferior cephalic oblique and posterior rectus major muscles.^5^, ^12^, ^13^ Unlike the previous approach, this technique can be performed using common instruments without the need for a dedicated minimally invasive channel. It provides a more flexible surgical field, but it may result in more damage to the trapezius, splenius capitis, and semispinalis capitis muscles compared to the minimally invasive channel approach.

### Anatomical Reference Point

The suboccipital triangle, which is bounded by the inferior cephalic oblique, posterior rectus major, and superior cephalic oblique muscles, played a crucial role in exposing the C1 lateral mass screw and C2 pedicle screw entry point. Careful treatment of the suboccipital triangle was necessary to ensure adequate exposure.^14^ In this study, the average area of the inferior occipital triangle was calculated to be 2.38 ± 0.59 cm^2^ providing an objective reference for the operating space in minimally invasive atlantoaxial fusion. By bluntly separating the soft tissues of the suboccipital triangle, we found that the posterior arch of C1 divides the suboccipital triangle into two parts, the superior part contains the horizontal segment of the vertebral artery that travels along the superior edge of the posterior arch of C1, and the inferior part contains the bony structures such as the C1 lateral mass and the C2 laminae that need to be revealed. However, the medial portion of the suboccipital triangle contains the vertebral canal and the lateral portion contains the vertical segment of the vertebral artery, so an anatomical reference point is needed to define the safe range between the medial and lateral sides during the separation.

During dissection, it was observed that the intersection of the occipital greater nerve with the inferior border of the inferior cephalic oblique was consistently present. The mean diameter of the greater occipital nerve was approximately 3.5 mm, making it easily identifiable intraoperatively.^17^ This intersection was identified as the anatomical reference point for minimally invasive posterior atlantoaxial fusion. Scherer et al.^19^ measured the mean distance from the C2 spinous process to the C1 transverse process, namely the mean length of the inferior cephalic oblique muscle, to be 5.60 ± 0.46 cm, and the mean distance between the C2 spinous process and the intersection of the greater occipital nerve on the inferior cephalic oblique muscle to be 3.56 ± 0.36 cm from 20 cadaveric specimens. The mean distance from the C2 spinous process to the C1 transverse process measured in this study was 47.66 ± 4.76 mm, and the distance from the C2 spinous process to the intersection of the greater occipital nerve and the inferior cephalic oblique muscle was 25.09 ± 3.53 mm. The differences in the measurements may be due to the different ethnicity of the source of the specimens, differences in measurement methods, and insufficient sample size. The distance from the C2 cspinous process to the C1 transverse process can be used as a specific anatomical parameter for each patient, providing an objective reference for assessing the distance between the atlas and the axis prior to minimally invasive atlantoaxial fusion surgery. The distance from the C2 spinous process to the intersection of the greater occipital nerve and the inferior cephalic oblique muscle can be used as a reference for the distance of the paracranial incision in minimally invasive surgery, and also as a reference for the site of the dissection of the splenius capitis and the semispinalis capitis muscle, namely, a minimally invasive incision or dissection of the splenius capitis and the semispinalis capitis muscle at about 2.5 cm from the midline can reveal the intersection of the greater occipital nerve and the inferior border of the inferior cephalic oblique muscle. The width and thickness of the inferior cephalic oblique muscle at the anatomical reference point can be used as a reference when pulling or detaching the inferior cephalic oblique muscle during the minimally invasive surgery. In this study, the C1 lateral mass screw entry point was measured at 18.13 ± 3.30 mm cephalad to the anatomic reference point, and the C2 pedicle screw entry point was measured at 4.23 ± 1.76 mm medial to the anatomic reference point. The relative positions of the anatomic reference point and the screw placement point can provide a guide for finding the C1 lateral mass screw and the C2 pedicle screw entry point after the anatomic reference point is revealed intraoperatively.

The anatomical reference point identified in this study is not a bony structure but rather a soft tissue landmark. This implies that its location may vary, particularly in cases of severe atlantoaxial dislocation or bony fusion dislocations. Therefore, the applicability of this reference point may be limited in such cases.^22^, ^23^ During minimally invasive surgery, the position of the anatomical reference point may change as a result of distraction or disconnection of the inferior cephalic oblique muscle. This can pose challenges in relying solely on the anatomical reference point for accurate placement of the C1 lateral screw and the C2 pedicle screw. To address these challenges, the study suggests using the distance between the anatomical reference point and the C1 lateral block screw placement point and C2 pedicle screw placement point. This approach allows the surgeon to locate the C1 lateral and C2 inferior articular regions and integrate bony reference markers for precise screw placement. By combining the anatomical reference point with bony landmarks, surgeons can enhance the accuracy and reliability of screw placement during minimally invasive atlantoaxial fusion procedures. However, it is important to note that further research and clinical validation are necessary to establish the effectiveness and safety of this approach in different patient populations and surgical scenarios.^24^, ^25^, ^26^

### Safety Range of Minimally Invasive Atlantoaxial Surgery

Protection of the vertebral artery is of utmost importance during atlantoaxial fusion surgery, as injury to this vessel can result in severe complications, including hemorrhage and even death. In this study, measurements were taken to determine the distances between the anatomical reference point and key structures related to the vertebral artery.^27^, ^28^, ^29^ The distance measured in this study was 9.14 ± 1.81 mm from the intersection of the occipital greater nerve and the inferior border of the inferior cephalic oblique muscle to the medial border of the C2 transverse foramen. This represents the shortest distance from the anatomical reference point to the vertical portion (V3 segment) of the vertebral artery. Additionally, the distance measured was 21.29 ± 1.93 mm from the intersection of the occipital greater nerve and the inferior border of the inferior cephalic oblique muscle to the superior border of the posterior arch of C1. This represents the shortest distance from the anatomical reference point to the horizontal segment of the vertebral artery. Based on these measurements, during minimally invasive atlantoaxial surgery, it is important to perform the procedure medial to the intersection of the occipital greater nerve and the inferior border of the inferior cephalic oblique muscle. This approach helps to avoid injuring the vertebral artery located lateral to the anatomical reference point, thus ensuring the safety of the procedure. By taking these anatomical considerations into account and adopting appropriate surgical techniques, surgeons can minimize the risk of vertebral artery injury during atlantoaxial fusion surgery.

Protection of the dura mater is crucial during posterior atlantoaxial fusion surgery to prevent complications such as cerebrospinal fluid leakage, neurological dysfunction, or paralysis. In this study, measurements were taken to determine the distance between the anatomical reference point and the spinal canal, which represents the location of the dura mater.^30^, ^31^ The distance measured in this study was 11.53 ± 2.18 mm from the intersection of the occipital greater nerve and the inferior border of the inferior cephalic oblique muscle to the spinal canal. This measurement provides important information for surgical planning and suggests that the surgeon should operate as close as possible to the inferior border of the inferior cephalic oblique muscle when exposing the C2 vertebral lamina. By operating in close proximity to the inferior border of the inferior cephalic oblique muscle, the surgeon can minimize the risk of damaging the dura mater, which is located medially and superiorly to the anatomical reference point. This approach helps to ensure the integrity of the dura and reduce the likelihood of complications associated with intraoperative dural injury.

### Limitation and Strengths

The limitation of this study is the small number of specimens used, which may affect the generalizability of the results. An anatomical study with a larger sample size would provide more representative data. Furthermore, while this study provides important anatomical measurements and proposes the intersection of the greater occipital nerve and the inferior border of the inferior cephalic oblique muscle as an anatomical reference point, further research is needed to verify the feasibility and safety of using this reference point in the context of minimally invasive atlantoaxial fusion procedures.

This study proposes the intersection of the greater occipital nerve and the inferior border of the inferior cephalic oblique muscle as the anatomical reference point in atlantoaxial minimally invasive surgery. Additionally, it measures the safety range of atlantoaxial minimally invasive surgery, providing a reliable reference for avoiding damage to the vertebral artery and the dura mater. The language used is clear, concise, and objective, with a formal register and precise word choice. This study lays a crucial groundwork for the advancement of minimally invasive atlantoaxial surgery with direct vision.

## Conclusions

The intersection of the greater occipital nerve with the inferior border of the inferior cephalic oblique muscle may provide a safe and reliable reference for minimally invasive posterior atlantoaxial surgery.

## Conflict of Interest Statement

All the authors declare that they have no conflict of interest.

## Ethics Statement

All authors listed meet the authorship criteria according to the latest guidelines of the International Committee of Medical Journal Editors, and all authors are in agreement with the manuscript.

## Author Contribution

Peirong Lian and Hu Chen: conceptualization, data curation, methodology and writing—original draft. Wanshun Wang and Changrong Zhu: methodology, validation and visualization. Qiang Tu and Xiangyang Ma: methodology and writing—review and editing. Hong Xia and Honglei Yi: conceptualization, funding acquisition, resources, supervision, and writing—review.
